# Dynamical organization of vimentin intermediate filaments in living cells revealed by MoNaLISA nanoscopy

**DOI:** 10.1042/BSR20241133

**Published:** 2025-02-12

**Authors:** Mariano Smoler, Francesca Pennacchietti, María Cecilia De Rossi, Luciana Bruno, Ilaria Testa, Valeria Levi

**Affiliations:** 1 Departamento de Química Biológica, Facultad de Ciencias Exactas y Naturales, CONICET – Universidad de Buenos Aires, Instituto de Química Biológica (IQUIBICEN), Buenos Aires, Argentina; 2 Department of Applied Physics and Science for Life Laboratory, KTH Royal Institute of Technology, 100 44, Stockholm, Sweden; 3 Facultad de Ciencias Exactas y Naturales, CONICET – Universidad de Buenos Aires, Instituto de Cálculo (IC), Buenos Aires, Argentina

**Keywords:** cytoskeleton, MoNaLISA nanoscopy, vimentin filaments

## Abstract

Intermediate filaments are intimately involved in the mechanical behavior of cells. Unfortunately, the resolution of optical microscopy limits our understanding of their organization. Here, we combined nanoscopy, single-filament tracking, and numerical simulations to inspect the dynamical organization of vimentin intermediate filaments in live cells. We show that a higher proportion of peripheral versus perinuclear vimentin pools are constrained in their lateral motion in the seconds time window, probably due to their cross-linking to other cytoskeletal networks. In a longer time scale, active forces become evident and affect similarly both pools of filaments. Our results provide a detailed description of the dynamical organization of the vimentin network in live cells and give some cues on its response to mechanical stimuli.

## Introduction

The cytoskeleton, a complex network formed by microtubules, actin, and intermediate filaments (IFs), allows cells to change shape, resist deformation, and organize internally [1,2]. Initial works in the area mainly focused on the function of actin and microtubule networks, but this trend changed in the last years due to a wealth of evidence showing the key roles played by IFs in a wide variety of cell processes [3–7].

IFs are intimately involved in the mechanical behavior of cells despite they are not responsible for force-generation events as microtubules or actin filaments. Assays with purified filaments revealed exquisite mechanical properties of IFs networks that seem to be fundamental for their biological functions. These experiments showed that IFs constitute the softest component of the cytoskeleton with small persistence lengths ranging from 0.2 μm to 3 μm according to the IF composition and the specific measurement conditions [8]. The elastic modulus of IF networks increases at large strains [9], and thus they withstand significantly greater mechanical deformation than actin and microtubules [10]. Indeed, IFs contribute to cell stiffness and protect the cell against mechanical stresses [11–13].

Vimentin is a type III intermediate protein widely studied for many years because the increment of its concentration during the epithelial to mesenchymal transition leads to changes in cell shape, motility, and adhesion [4,11]. Therefore, this protein is considered a biomarker of increased metastasis [14] highlighting the relevance of understanding its biological roles.

Vimentin IFs interact with the microtubule and actin networks through protein cross-linkers, molecular motors, and other structures such as the plasma membrane and organelles [15–20] to accomplish many cellular functions involving the cytoskeleton. For example, the interaction of vimentin filaments with the actin cortex is required to achieve normal mitosis [21,22]. In addition, the vimentin network constitutes a template for microtubule organization during cell migration [23] and the interactions of this network with actin control cell mobility [18]. Furthermore, we showed that the apparent persistence length of vimentin filaments determined in living cells (lp*) as well as the lateral dynamics of these filaments is modulated by the actin and microtubule networks evidencing that the mechanical communication with other cytoskeletal filaments also affect vimentin filaments [24]. Unfortunately, the low spatial and temporal resolutions of these previous measurements did not permit the extraction of quantitative information about the processes involved.

Complementing these observations, we found that the elastic vimentin network modulates the apparent persistence length of microtubules and mechanically reinforces them in living cells [25]. These simple examples illustrate the constant mechanical communication between the cytoskeletal networks within the cell.

Vimentin filaments form a cage-like structure surrounding the nucleus in many cell types [26]. This structure protects the organelle from mechanical stresses and is fundamental to prevent its fragmentation and DNA breakage during cell migration [27]. Noteworthy, the cage does not seem to include microtubules or actin filaments [26] which seems surprising given the interactions of vimentin filaments with the other cytoskeletal networks. Consequently, Patteson et al. [26] proposed distinct properties and functional roles of the perinuclear and cytoplasmic vimentin pools. Unfortunately, a deep understanding of vimentin organization in live cells is limited by the optical resolution of conventional fluorescence microscopy (~200 nm) which does not allow observing details of the highly intermingled vimentin network.

Here, we examine relevant aspects of the vimentin network organization in living cells by using Molecular Nanoscale Live Imaging with Sectioning Ability (MoNaLISA), a super-resolution microscopy approach based on the reversible saturable/switchable optical fluorescent transition (RESOLFT) concept that allows time-lapse imaging of whole cells with resolution of ~50 nm and reduced photodamage and photobleaching [28]. The ~1 Hz acquisition time provided by the parallelized reading strategy allowed us to study the dynamics of single vimentin filaments with nanometer precision and high temporal resolution. Our results show that perinuclear vimentin filaments present less-restricted lateral mobilities in comparison with peripheral filaments in a time window of seconds. In the minute time scale, both pools of filaments present anomalous diffusion with coefficients ranging from sub- to super-diffusion. Airyscan microscopy observations and numerical simulations support that a combination of passive processes and active forces produced by actin filaments and microtubules modulates the dynamics of vimentin filaments. Taken together, our results reveal new aspects of the dynamical organization of the vimentin network in live cells and give clues to understand its response to mechanical stimuli.

## Methods

### Cell culture and transfection

We used U2OS cells endogenously expressing vimentin-rsEGFP2 previously generated by Ratz et al. [29]. These cells were cultured in Dulbecco’s modified Eagle medium (Thermo Fisher Scientific, 41966029) supplemented with 10% (v/v) fetal bovine serum (Thermo Fisher Scientific, 10270106) and 1% (w/v) penicillin-streptomycin (Sigma Aldrich, P4333) and maintained at 37°C and 5% CO_2_ in a humidified incubator. For imaging, 2 × 10^5^ cells per well were seeded on 18-mm coverslips (No1.5) and imaged after 24–48 h. The nuclei were labeled with SiR-Hoechst following the producer protocol (SiR-DNA kit, Spirochrome). In particular, SiR-Hoechst (2 µm) and verapamil (10 µm) were added to the cell culture media for an incubation time of 30 min to 1 h. Cells were then washed, and imaging was performed in Leibovitz media supplemented with 10% (v/v) fetal bovine serum (Thermo Fisher Scientific, 10270106) and 1% (w/v) penicillin-streptomycin (Sigma Aldrich, P4333). For the control, cells were fixed with 4% (w/v) PFA for 15 min, washed three times with PBS, and then immediately recorded in a time-lapse acquisition.

Airyscan experiments were performed using U2OS cells transiently transfected with the following plasmids: GFP-vimentin [30] was provided by Dr. Vladimir I Gelfand (Northwestern University, Chicago, IL); mCherry-vimentin-7 (Addgene # 55156); EMTB-3xGFP [31], which codifies the microtubule-binding domain of ensconsin fused to a tandem of three copies of GFP (Addgene # 26741), kindly provided by Dr. Arpita Upadhyaya (University of Maryland, College Park, MD); pEGFP-actin [32] was a gift from Dr. Nicolás Plachta (Institute of Molecular and Cell Biology, ASTAR, Singapore); and PGK-H2B-mCherry [33] provided by Mark Mercola (Addgene plasmid # 21217). Cells were transfected with Lipofectamine 2000 (Thermo Fisher) and imaged 24 h after.

### MoNaLISA microscopy

We used a homemade MoNaLISA setup as reported in Masullo et al. [28]. All the images have been acquired with a multifoci pattern of periodicity 625 nm coupled with an OFF pattern of 312.5 nm. For rsEGFP2 imaging, the ON switch is performed with 405 nm light at 650 W/cm^2^ for 0.5 ms, the OFF confinement is performed with 1.5 ms of 488 nm light at 650 W/cm^2^, and finally the readout is recorded over 1 ms of illumination of 488 nm at 240 W/cm^2^. The step size and therefore the pixel size is 35 nm, for a global dwell time of 5 ms and recording time of 1.94 s. The image stacks were recorded either without waiting time between frames or with a time interval of 30 s as described in the first and second sections of Results. Before the acquisition of MoNaLISA movies of vimentin, we acquired two confocal images of the nuclei, one at the same optical plane of the recorded vimentin movie and the other ~ 1–2 μm above this plane. The images of nuclei were recorded using 350 W/cm^2^ of 590 nm light with a time frame of 4.9 s. For imaging the SiR emission, a filter at 670/40 nm has been used on a second camera. The alignment between the two cameras was achieved with a reference sample composed of disperse beads with a wide fluorescent spectrum (TetraSpeck Microsphere, 0.1 µm, T7279, Thermo Fisher).

All the reported images are raw data. To help the comparison, we applied a Gaussian smoothing of 20 nm to the data shown in the first figure. The cells did not move significantly during the acquisition time, as assessed from the comparison of the first and last frame of each movie Microscopy experiments were performed at room temperature.

### Airyscan microscopy

We acquired time-lapse images in a Zeiss LSM980 confocal microscope (Carl Zeiss) equipped with the Airyscan 2 detector using a Plan-Apochromat 63× oil immersion objective (NA = 1.4). EGFP (or GFP) and mCherry fluorescent proteins were excited with solid diode lasers of 488 nm and 543 nm, respectively. Movies were registered at ~ 0.5 frames/s with a pixel size of 43 nm.

### Single filament tracking with nanometer resolution

The coordinates of IFs segments were recovered from MoNaLISA images using the single-filament tracking routine AFTER [25]. Briefly, the program applies a rotation transformation to the image in such a way that the x_k_ coordinates of the filament are univocally associated with single y_k_ values. For each x_k_, the algorithm extracts the intensity profile in the vertical direction and localizes the filament center with nanometer precision using a generalized regression neural network [34,35]. AFTER outperforms the traditional Gaussian deconvolution methods at the signal-to-noise ratios observed for cytoskeletal filaments in living cells, thus providing the filament coordinates with smaller errors [25].

### Fourier decomposition of filament shapes

We used the filament coordinates (x_k_, y_k_) recovered from the images to calculate tangent angles:


(1) $$\theta_{k}={tan}^{-1}\left(\frac{y_{k+1}-y_{k}}{x_{k+1}-x_{k}}\right)$$

and segment lengths:


(2) $$S_{k}=\sqrt{{\left(x_{k+1}-x_{k}\right)}^{2}+{\left(y_{k+1}-y_{k}\right)}^{2}}$$

The tangent angle is then decomposed into a large number of Fourier modes and expressed as a sum of cosines [36]:


(3) $$\theta \left(s\right)=\sqrt{2/L}\sum_{n=0}^{\infty }\;a_{n}cos\left(q_{n}s\right)$$

where q_n_ represents the wave vector defined as $q_{n}=n\pi /L$. The zero-order term a_0_ corresponds to the average orientation of the filament, s is the arc length along the filament, and L is the segment length.

### Determination of the apparent persistence length

The persistence length of filaments can be obtained by analyzing the shapes of the filaments through a Fourier decomposition procedure [36]. Recent works [25,36] also showed that the curvatures of vimentin filaments and microtubules in living cells followed a thermal-like distribution characterized by:


(4) $$ln\left(Var\left(a_{q}\right)\right)=-ln\left(lp^{*}\right)-2ln\;q$$

where (Var(a_q_)) is the variance of the Fourier mode amplitudes averaged over uncorrelated filament’s shapes, q is the mode q_n_, and lp* is the apparent persistence length.

We fitted equation 4 to the Fourier data; the standard error (SE) of lp* was obtained from the propagation of the intercept’s error. lp* values were considered different if their lp* ± SE intervals did not overlap.

### Trajectory analyses

The length of tracked filament’s segments ranged between 0.5 and 6.5 µm. The lateral position of each segment was computed as its mean y coordinate (Y_CM_) and the mean lateral square displacement (MSD_L_) was calculated as [25]:


(5) $$MSD_{L}\left(\tau \right)=\left\langle {\left(Y_{CM}\left(t\right)-Y_{CM}\left(t+\tau \right)\right)}^{2}\right\rangle$$

where t and τ are the absolute and lag times, respectively, and the brackets represent the time average. This calculation was done for τ< 50% of the total time of the trajectory.

Those MSD_L_ versus τ curves presenting confined motion were individually fitted with the following equation that assumes corralled diffusion [37]:


(6) $$MSD_{L}\left(\tau \right)=r_{c}^{2}\left(1-e^{\frac{-\tau }{\tau_{c}}}\right)+MSD_{0}$$

where τ is the time lag, r_c_ is the radius of the corral, τ_c_ is a characteristic time, and MSD_0_ is a residual term

The parameters obtained from the fitting of equation 6 were used to estimate the lateral diffusion coefficient of the filament (D) within the corral [37]:


(7) $$D=\frac{r_{c}^{2}}{2\tau_{c}}$$

Those MSD_L_ curves presenting anomalous diffusion are fitted with the following equation [38]:


(8) $$MSD_{L}\left(\tau \right)=D_{app}{\left(\frac{\tau }{\tau_{0}}\right)}^{\alpha }+MSD_{0}$$

where τ is the time lag, τ_0_ is a constant set to 1 s, D_app_ is a pre-exponential factor, and α is the anomalous diffusion exponent. MSD_0_ is a residual term related to the tracking error and to motion of the filament faster than the sampling speed [38]; in the analyzed time window (1–4 min), $MSD_{0}\ll D_{app}{\left(\frac{\tau }{\tau_{0}}\right)}^{\alpha }$, and thus we considered this residual term negligible.

### Numerical simulations

We simulated 100 trajectories during 600-s total time with a time step (dt) of 10^−4^ s for each set of parameters values (Supplementary Table S1) using home-made MATLAB (The Math Works) routines. The simulated trajectories were resampled to a frequency of 30 Hz in line with the experiments described in the second section of Results.

### Statistical analysis

Statistical significance among data sets median values (med) were analyzed using a hypothesis test computing the *P* values as follows [39]:


(9) $$p\text{-value}=2\left[1-F\left(\frac{\left|med_{\left(g1\right)}-med_{\left(g2\right)}\right|}{\sqrt{\sigma_{\left(g1\right)}^{2}+\sigma_{\left(g2\right)}^{2}}}\right)\right]$$

where F is the standard normal distribution and σ^2^ (g1) and σ^2^ (g2) represent the variance of each data group. Differences were considered significant if *P*<0.05.

The parameters’ errors and variance were computed by a bootstrap procedure [40].

Statistical differences among distributions were analyzed by applying a two-sample Kolmogorov-Smirnov test. Differences were considered significant if *P*<0.05. Statistical data analysis was performed using Phyton routines.

## Results

### The lateral motion of vimentin filaments is constrained in a time scale of seconds

**Figure 1:**
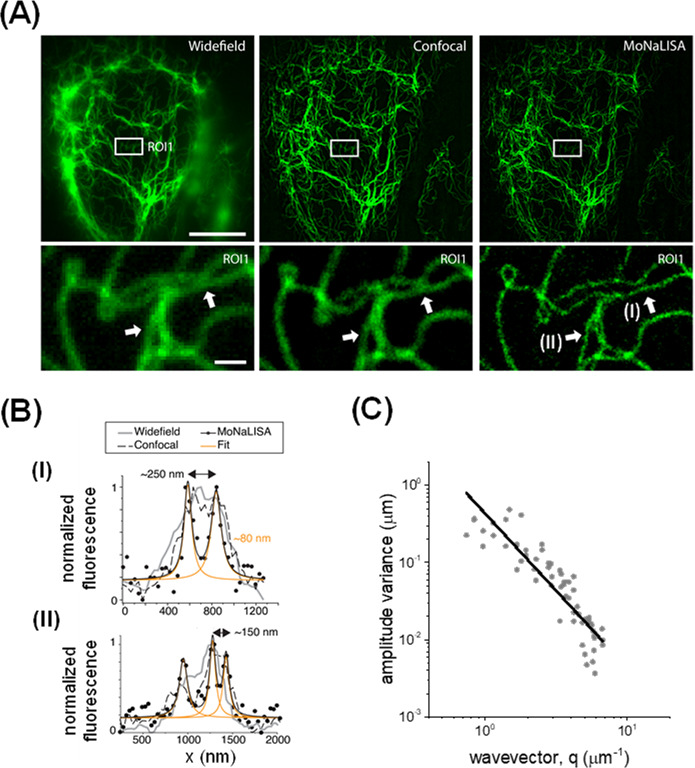
Determination of the apparent persistence length of vimentin filaments in living U2OS cells. **(A)** Representative images of U2OS cells endogenously expressing rsEGFP2-vimentin acquired with widefield, confocal and MoNaLISA setups (upper panels). Zoom-in images of the regions included in the rectangles delimited in the whole-cell images (bottom panels). Scale bars: 10 µm and 1 µm, respectively. **(B)** Intensity profiles perpendicular to the main axis of close-by vimentin filaments (arrows); the filaments width (~ 80 nm) in the MoNaLISA image was determined by fitting a Lorentzian function (orange lines) to the intensity profiles (black dots). **(C)** Analysis of filament´s curvatures by a Fourier decomposition method. The continuous line corresponds to the fitting of equation 4 to the Fourier data.

We acquired MoNaLISA images of U2OS cells expressing endogenous levels of vimentin fused to the reversibly switchable fluorescent protein rsEGFP2 (rsEGFP2-vimentin) [29]. In line with previous results [28], Figure 1A-B shows that this nanoscopy allows observing subtle details of the vimentin network that remain undetectable in widefield and confocal imaging.

In order to analyze the performance of this microscopy in the study of single vimentin filaments in living cells, we first quantified their lp*; this parameter provides information on the mechanical properties of cytoskeletal filaments in cells and their mechanical crosstalk with other components of the cytoskeleton [24,25,36]. We used the filament tracking routine AFTER to recover the coordinates of filament’s sections with subpixel resolution [25] and analyzed the curvatures of the filaments using the Fourier decomposition procedure described before [24]. The lp* value obtained by fitting equation 4 to the experimental data (Figure 1C) was 2.3 ± 0.2 μm; this value is not significantly different from those obtained previously in other cell lines (BHK and transformed and metastasizing BJ fibroblasts) imaged with confocal and STED microscopies (~2.2 μm [24]).

To study the dynamics of the perinuclear and peripheral pools of vimentin filaments, we evaluated their lateral mobility in cells also labeled with SiR-Hoechst to visualize the nucleus. We acquired MoNaLISA images of the vimentin network in whole, single cells at a speed of 0.5 frames/s during 50 s. Immediately before this time-lapse acquisition, we collected confocal images of SiR-Hoechst labeling in the basal plane of the same cells and ~1–2 μm above this plane (i.e. in the plane that gives the largest nuclear area) as MoNaLISA requires the photoswitchable probe and does not allow observing the nuclear probe.

**Figure 2:**
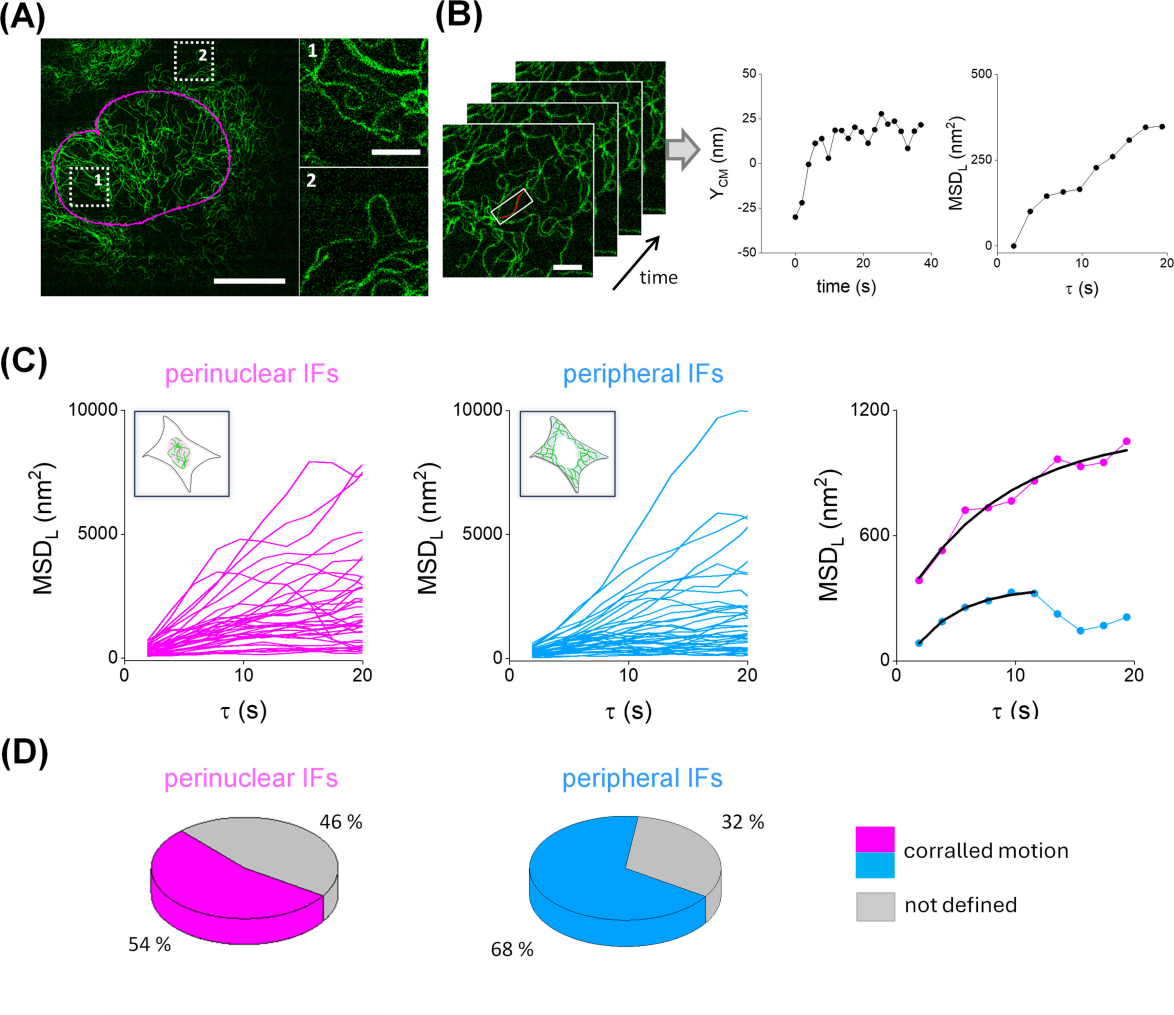
The lateral motion of vimentin filaments followed a constrained diffusion model in a time window of seconds. **(A)** Vimentin IFs were classified as perinuclear and peripheral (dotted squares:1 and 2, respectively) based on their location relative to the contour of the cell nucleus (magenta line) as described in the text. Scale bar: 10 µm. Zoom-in regions of the image, to facilitate the visualization of individual filaments. Scale bar: 2 µm. **(B)** Procedure for the quantitative analysis of single filament trajectories. The spatial coordinates of the segments were recovered in each frame of the movie to obtain the trajectory of its center of mass (Y_CM_) and the MSD_L_. Scale bar: 2 µm. **(C)** MSD_L_ curves obtained for perinuclear (magenta) and peripheral (light blue) vimentin filaments showing corralled motion. The data were individually fitted with equation 6 for τt < τt_corral_ (black line). **(D)** Proportion of corralled motion observed in each subcellular region.

We arbitrarily classified the filaments as perinuclear or peripheral according to their relative position with respect to the nucleus (Figure 2A). Specifically, we segmented the nucleus in the upper plane and considered filaments as perinuclear if they were located inside this nuclear perimeter. U2OS cells are relatively thin when attached to glass substrate, and thus, the procedure allows distinguishing those vimentin filaments in close proximity to the bottom region of the nucleus from those in the periphery (Supplementary Figure S1).

Next, we used AFTER to recover the coordinates of single filament segments in each frame of the movie (Figure 2B). We obtained the trajectories of their mean lateral position (Y_CM_) and computed the MSD_L_ as described in Methods. Additionally, we calculated the deformation index (DI) for each segment defined as the ratio between the mean end-to-end distance in the image-stack and its contour length. This index changed <10% (Supplementary Figure S2), suggesting that the analyzed segments minimally deform within the assayed time window. Finally, we determined that the precision of the localization of single vimentin filaments was ~15 nm by running similar experiments in fixed cells (Supplementary Figure S3).

We visually inspected the MSD_L_ of single filaments in live cells and observed that most of the trajectories followed a corralled motion characterized by the increase in MSD_L_ until reaching a plateau at a characteristic τ value that depends on the trajectory (Figure 2C). This behavior is expected for filaments anchored to or constrained by other structures that remain static in the studied time window; in these cases, there are no forces acting on the vimentin filaments that promote their displacement outside the confined region.

Those MSD curves presenting confined motion were individually fitted with equation 6 that assumes corralled diffusion; Table 1 and Supplementary Figure S4 compile the median values of the fitting parameters and their distributions, respectively. Filaments spent ~ 17–20 s in the corralled behavior (τ_corral,_ Table 1), and after the plateau, the MSD_L_ curves show a variety of behaviors probably associated with other intracellular processes that alter the dynamics of the filaments. Transitions between different mobility regimes are common inside the cell, due to a wide variety of active and passive processes spanning different time scales [41].

**Table 1:** Median values of the parameters obtained for the corralled diffusion model. The data are expressed as median ± standard error

Subcellular region	N_tracks/_N_cells_	τ_corral_ (s)	r_c_ (nm)	τ_c_ (s)	MSD_0_ (nm^2^)
Perinuclear	41/9	17.5 ± 0.4	47.4 ± 5.6	8.8 ± 1.4	−213 ± 60
Peripheral	35/9	19.4 ± 1.3	41.6 ± 4.8	7.7 ± 1.5	−174 ± 50

The parameters in the perinuclear and peripheral regions were not significantly different suggesting that the intrinsic mobility of corralled filaments in this time window is similar in both regions. On the other hand, we estimated the corral diameter in ~80 nm (Table 1); considering that the diameter of a vimentin filament is ~11 nm [42], the lateral movement of the filament is constrained to a short distance equivalent to ~7 times the filament diameter.

Relevantly, we verified that a higher proportion of peripheral filaments followed corralled motion (Figure 2D) suggesting that more vimentin filaments anchored to static cytoskeleton structures in the periphery in comparison to the perinuclear region. Considering that the lateral movements of microtubules are in a longer time scale (10–100 s [43]) and that vimentin filaments are transported along microtubules toward the peripheral region [26], one possible explanation for our results is that vimentin filaments cross-link in a higher proportion with nearby microtubules in the peripheral region.

### Influence of active intracellular processes on the lateral mobility of vimentin filaments

To get further insights on the interactions between vimentin filaments and the active networks of the cytoskeleton, we first inspected qualitatively the physical coupling of single vimentin filaments with the microtubule and actin cytoskeleton using Airyscan microscopy. Although it improves the spatial resolution by a factor of ~1.7 with respect to confocal microscopy [44], it does not reach MoNaLISA’s resolution, thus limiting our observations to regions with relatively low densities of filaments.

In these experiments, we used U2OS cells transiently expressing mCherry-vimentin and EMTBx3GFP, which binds to microtubules, or EGFP-actin. Supplementary Figure S5 shows that the overall organization and persistence length of vimentin filaments in these cellular system and imaging conditions are not significantly different from that determined before (Figure 1C and [24]).

**Figure 3:**
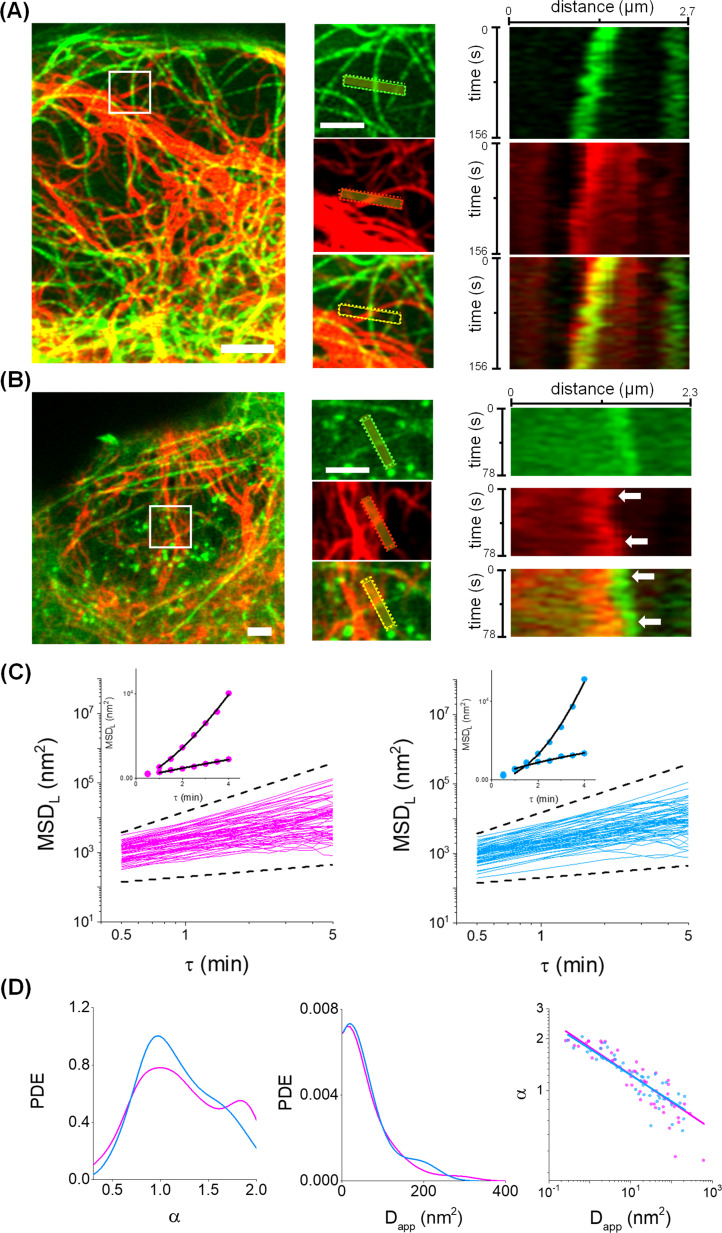
The lateral motion of vimentin filaments followed anomalous diffusion in a time window of minutes. **(A-B)** Representative Airyscan images of U2OS cells co-transfected with mCherry-vimentin (red) and EMTBx3GFP (**A**, green) or EGFP-actin (**B**, green). Scale bar: 2 µm. Zoom in regions (white squares, left panel) and kymographs (right panel). The arrows point contact sites between filaments. Scale bar: 2 µm. **(C)** MSD_L_ curves presenting anomalous diffusion obtained for perinuclear (magenta) and peripheral (light blue) filament segments in log-log scales. Dotted lines display the expected behavior for sub diffusion with α=0.5 (bottom) and ballistic motion (α=2, top). DI values determined for these tracking data were < 10%. Inset: representative MSD_L_ data fitted with equation 8 (continuous line). **(D)** Distributions of α and D_app_ obtained by fitting individual MSD_L_ data (left and middle panels) and their relation in log-log scale (right panel). Continuous lines correspond to the fitting of an allometric function, $\alpha =A\cdot {D_{app}}^{B}$, where A and B are constants.

Figure 3A-B shows representative two-color images and kymographs obtained by Airyscan microscopy revealing coupled motions of vimentin filaments with microtubules or actin, suggesting an active, physical communication between vimentin and the other two networks in the time scale of these experiments (78–156 s).

We next used MoNaLISA to quantitatively evaluate the dynamics of vimentin filaments in a time window in which is evident the coupling with the other active cytoskeletal networks. The image stacks were recorded with a time interval of 30 s and analyzed as described previously to recover the trajectories of the filaments and their MSD_L_.

The examination of these data indicated that a similar proportion of filaments (80% and 76% of the perinuclear and peripheral filaments, respectively) followed anomalous diffusion (Figure 3C). Therefore, we fitted equation 8 to the single MSD_L_ data in the τ range of 1–4 min and computed both the anomalous diffusion exponent (α) and the pre-exponential term (D_app_). Table 2 and Figure 3D show that the median values of α and D_app_ and their distributions were not significantly different for perinuclear and peripheral filaments.

**Table 2:** Median values of the fitting parameters obtained for the anomalous diffusion model. The data are expressed as median ± standard error

Subcellular region	N_tracks/_N_cells_	α	D_app_ (nm^2^)
Perinuclear	45/9	1.1 ± 0.1	18 ± 5
Peripheral	44/10	1.1 ± 0.1	20 ± 7

We next asked if the arbitrary classification of filaments as perinuclear or peripheral could hide a particular spatial distribution of the parameters and built cellular maps of D_app_ and α (Supplementary Figure S6). These maps did not show an evident correlation between the position of the filaments and the parameter values, suggesting that the observed variability is not related to the relative position of the filaments inside the cell.

Figure 3D shows that the anomalous diffusion exponent presents a broad distribution in the range 0.5–2, approximately. This parameter provides information on the overall directionality of the trajectory, and thus it is related to the balance of active versus passive forces. On the other hand, the pre-exponential term D_app_ informs on the motion amplitude and is proportional to the diffusion coefficient in Brownian diffusion and to the velocity squared for ballistic motion [45].

The broad distribution of α, spanning from sub- to super-diffusion, suggests that vimentin filaments are immersed in heterogeneous microenvironments and exposed to forces that probably act transiently, interspersed with periods of thermal jittering constrained by the cross-linking to other structures as discussed in the previous section.

Additionally, Figure 3D shows that those trajectories presenting high directionalities (i.e. high values of α) also show low amplitude of motion (i.e. low values of D_app_). This dependence is not expected for a pure process and could be a consequence of the competition between different processes acting on the filaments in this time scale.

### The dynamics of intermediate filaments in a time scale of minutes revealed a competition between active versus passive processes

We run numerical simulations to better understand the observations performed in the previous section and more specifically the relation between the parameters characterizing the anomalous diffusion observed in Figure 3D .

**Figure 4:**
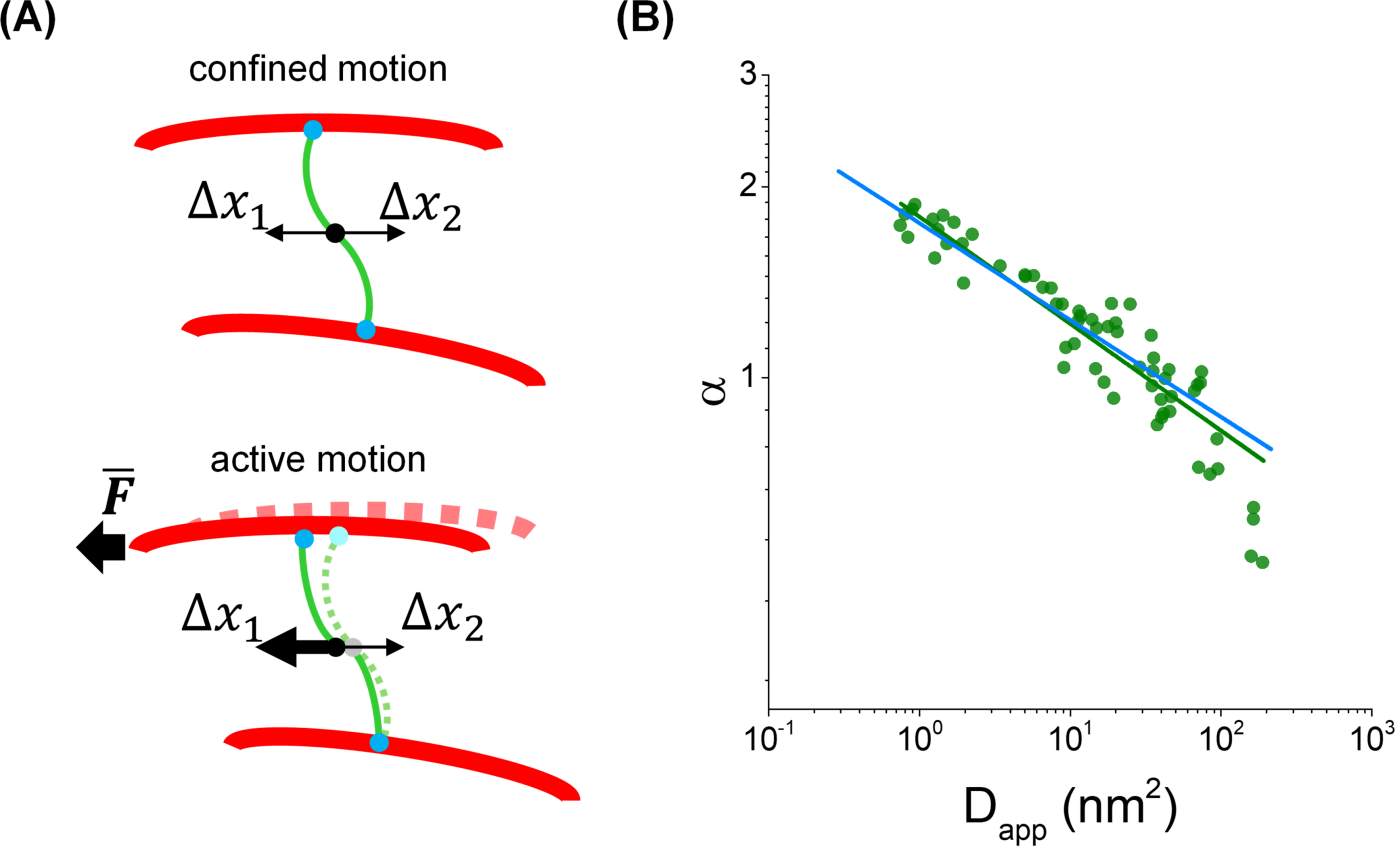
Numerical simulations of the lateral dynamics of vimentin filaments. (**A**) Cartoon illustrating the simulated model. In a time scale of seconds, the absence of net external forces acting on vimentin filaments (green) generates displacements in a confined space ($\Delta x_{1}=\Delta x_{2}$) due to its connections to static cytoskeletal polymers (red). Active forces transmitted by the cytoskeleton become appreciable at longer periods of time and indirectly affect the mobility of the vimentin filaments ($\Delta x_{1}§gt;\Delta x_{2}$). The black arrow illustrates a net displacement of a cytoskeletal polymer in the horizontal direction. (**B**) Relation of α and D_app_ parameters recovered from the simulations (green). Continuous lines represent the fitting of the simulated (green) and peripheral (light blue, Figure 3D) data with an allometric function $\alpha =A\cdot {D_{app}}^{B}$, where A and B are constants.

A simple model (Figure 4A)is proposed that considers the motion of a particle representing the center of a filament laterally diffusing within a confined space (i.e. a corral determined by the cross-linking of the filament to other cytoskeletal filaments). The particle is also subjected to transient, directional forces (those produced by the cross-linked microtubules or actin filaments). Mathematically, the particle performs a unidimensional random walk with steps of size *l*_step_ within a corral of dimension r_c_. After a given period of time (τ_corral_), the particle is allowed to move directionally performing a jump (*l*_jump_) longer than *l*_step_ and with direction drawn with probability *p*_jump_. The simulation ends at 600 s, i.e. the time window of the experiments shown in the previous section.

Supplementary Table S1 summarizes the ranges of parameter values used for these simulations. Specifically, r_c_ and τ_corral_ were similar to those derived from the experiments and listed in Table 1. Each time the particle leaves a corral, new r_c_ and τ_corral_ values are randomly sampled from the experimental distributions. *l*_step_ was obtained using the lateral diffusion coefficient of the filament in the corral, estimated from the experimental data (Supplementary Figure S7). On the other hand, we scanned p_jump_ and *l*_jump_ in a wide range of values until the simulated trajectories adequately represented the experimental results.

The simulated trajectories were analyzed as described before; specifically, we calculated the MSD_L_, inspected the curves individually, and fitted them to the anomalous diffusion model. Some of the simulated trajectories presented a confined behavior (~ 30 %) and were discarded in further analyses. Supplementary Figure S8A compiles some of the simulated and experimental trajectories and their respective MSD_L_ to facilitate the comparison between the experimental and simulated data.

Supplementary Figure S8B shows that the parameters obtained by fitting the simulated MSD_L_ with equation 8 are distributed similarly to the experimental parameters. In particular, the simulations allowed recovering the broad distribution of α observed in the experiments. The dependence of D_app_ with α (Figure 4B) was similar to that obtained for the experimental data suggesting that the model could explain the lateral mobility of vimentin filaments in this time scale.

## Discussion

IFs play fundamental roles in a wide variety of cellular processes including the mechanical communication of external mechanical cues to the cell interior and, particularly, to the cell nucleus. These signals trigger responses ranging from changes in the cell shape to modifications in the gene expression profile. Rather than simply defining the viscoelasticity of the cytoplasm, it is becoming increasingly evident that the functions of IFs include, but are not limited to, this simple role.

Many of these properties rely on a continuous crosstalk between the cytoskeleton networks, each one formed by biopolymers with different biophysical properties such as the persistence length and the response to strain that define synergistic composite properties fundamental for the mechanical behavior of cells [46].

Hu et al. [47] showed that vimentin filaments behave as a strain-stiffening hyperelastic network, in contrast with microtubules and actin filaments which disassemble at relatively low strains. This observation highlights the exquisite biophysical properties of the vimentin filaments that could be fundamental to disperse local deformations in the cytoplasm when cross-linked to other cytoskeletal networks preventing cell damages [47].

Early observations distinguished two different architectural motifs of the vimentin network, the perinuclear cage composed of an intricate basket-like structure surrounding the nucleus and the peripheral network, with filaments dispersing throughout the cytoplasm and reaching the cell borders [26]. Also, it was reported that the stiffness of cells is not homogeneous and increases toward the periphery [48] probably due to heterogeneities in, for example, the distribution and mechanical coupling of the vimentin network with the other cytoskeletal networks. Unfortunately, the standard methodologies in the field did not allow exploring these heterogeneities with high spatial resolution in living cells.

Here, we analyzed the properties of vimentin filaments using MoNaLISA nanoscopy that allows acquiring whole cell images and tracking single vimentin filaments with ~15 nm precision and reduced photodamage.

Our experiments revealed that many vimentin filaments display corralled motion in a time scale of seconds probably due to their cross-link to other cytoskeleton components (i.e. microtubules and actin filaments) or structures that remain stationary in this time scale [41,43]. Importantly, the proportion of filaments undergoing confined motion is higher in the peripheral region suggesting a higher proportion of cross-linked vimentin filaments in this zone, which could play a relevant role in reinforcing the cytoskeleton mechanically to support the higher traction stresses produced in the cell periphery [49]. In line with this statement, we have previously observed that microtubules in the cell periphery curve up in the absence of the vimentin network showing that these last filaments mechanically reinforce microtubules [25]. In addition, previous data suggest that the nuclear cage is not particularly enriched in microtubules or actin filaments [26]. The small size of the confinement region (~80 nm diameter) also suggests a tight connectivity of vimentin filaments with the cytoskeleton and/or other cytoplasmic structures.

We also analyzed the lateral motion of single vimentin filaments in a time scale of minutes. As observed through Airyscan microscopy, active processes involving the motion of microtubules and actin filaments become evident in this longer time window, and thus, they are probably the main generators of forces that affect the mobility of vimentin filaments.

We found that the lateral motion of vimentin filaments could be explained by an anomalous diffusion model with similar α and D_app_ values in the perinuclear and peripheral regions. These results suggest that, in this time scale, the coupling of vimentin filaments with the other cytoskeletal networks is similar in both regions. Relevantly, the broad distribution of α values spanning from sub- to super-diffusion suggests a complex and heterogeneous dynamics of vimentin filaments. Moreover, the observed correlation between α and D_app_ values also supports that a combination of different dynamical processes drives the motion of these filaments.

Finally, we integrated the dynamical information in both time windows with a reduced and simple model that considers the corralled motion of the filaments coupled to the transient, active transport of the filaments. The numerical simulations reproduced sufficiently well the experimental correlation between αand D_app_ values.

Costigliola et al. [50] showed that the vimentin network presents mesh-like regions and fibers formed by bundles of filaments and suggested that the former structures contribute to the cell response to traction stresses. Our analyses, focused on single filaments belonging to the mesh-like regions, suggest that the meshes could be also modulated by mechanical stress. We speculate that those mechanical signals received by the cell or generated in the cell cytoplasm could be communicated more efficiently to other mechanically coupled regions of the peripheral cytoplasm in comparison with the perinuclear regions. This differential transmission could be relevant to prevent short and brief mechanical stimuli from reaching the nucleus. In a longer temporal scale (i.e. minutes), the dynamics of peripheral and perinuclear filaments are similar suggesting that the response of the IF network to persistent mechanical stimuli produced in this time window will be similar in both regions. We should also mention that it is scientifically challenging to directly associate the dynamical properties of vimentin filaments in the second-to-minute time scale described in our work with cell processes occurring in a significantly longer time scale such as cell migration, a process that also involves numerous cellular components beyond the vimentin network. Future studies on this topic will be needed to experimentally test this hypothesis.

## Supplementary material

Figure S1

Figure S2

Figure S3

Figure S4

Figure S5

Figure S6

Figure S7

Figure S8

Table S1

## Data Availability

All relevant data are included within the manuscript and its Supplementary information files.

## Abbreviations

DI: deformation index
IFs: intermediate filaments
MSD_L_: mean lateral square displacement
MoNaLISA: Molecular Nanoscale Live Imaging with Sectioning Ability
RESOLFT: reversible saturable/switchable optical fluorescent transition
SE: standard error
lp*: apparent persistence length
