# Supplementary material for: Dynamical organization of vimentin intermediate filaments in living cells revealed by MoNaLISA nanoscopy
Source: Biosci Rep. 2025 Feb 12;45(2):BSR20241133. doi: 10.1042/BSR20241133 (PMC12127793; doi:10.1042/BSR20241133)
Supplement: Figure S1 [file bsr-45-02-bsr-2024-1133-s001.docx]

**Supplementary Figure S1.** Representative 3D image of a U2OS cell expressing rsEGFP2-vimentin. (**A**) Overlay between the confocal image of the nucleus (magenta) and the MoNaLISA image of the vimentin network (green). Box: 40 x 40 x 5 μm^3^. (**B**) Single-plane of the image´s overlay at 1 µm of the bottom plane of the cell. The yellow lines in the z-projections show the plane used for defining the perinuclear and peripheral regions. To facilitate the visual observation of the network, we applied an intensity threshold to this image, which was also digitally saturated.
