# Supplementary material for: Dynamical organization of vimentin intermediate filaments in living cells revealed by MoNaLISA nanoscopy
Source: Biosci Rep. 2025 Feb 12;45(2):BSR20241133. doi: 10.1042/BSR20241133 (PMC12127793; doi:10.1042/BSR20241133)
Supplement: Figure S2 [file bsr-45-02-bsr-2024-1133-s002.docx]

**Supplementary Figure S2.** Representative trajectory of the deformation index. (**A**) Spatial coordinates of an individual filament segment recovered in 5 frames of the time-stack. (**B**) Trajectory of the deformation index (DI) for the filament segment shown in (A).  The contour length of the segment was recovered in each frame of the stack, divided by the average length obtained in the movie and normalized by the mean DI determined for the trajectory. DI variation was < 10% along the stack duration. The red dashed lines indicate the tolerance limit established to include the filament segment in subsequent analyses.
