# Supplementary material for: Dynamical organization of vimentin intermediate filaments in living cells revealed by MoNaLISA nanoscopy
Source: Biosci Rep. 2025 Feb 12;45(2):BSR20241133. doi: 10.1042/BSR20241133 (PMC12127793; doi:10.1042/BSR20241133)
Supplement: Figure S3 [file bsr-45-02-bsr-2024-1133-s003.docx]

**Supplementary Figure S3.** Precision on the localization of single vimentin filaments. (**A**) Representative image of a region of a fixed U2OS cell expressing rsEGFP2-vimentin. The white asterisk indicates a filament region selected for tracking. Scale bar: 2 µm. (**B**) Tracking experiments were performed as described for living cells. Spatial coordinates recovered in some frames of the movie for the filament shown in (A). The precision on the filament position determination (~ 15 nm) was obtained from the MSD_L_ analysis of 45 filaments (N_cells_ = 5) following a procedure similar to that described previously [1].

**Supplementary references**

1. Martin DS, Forstner MB, Kas JA. (2002) Apparent subdiffusion inherent to single particle tracking. *Biophys J*.**83**(4), 2109-17. https://doi.org/10.1016/S0006-3495(02)73971-4.
