# Supplementary material for: Dynamical organization of vimentin intermediate filaments in living cells revealed by MoNaLISA nanoscopy
Source: Biosci Rep. 2025 Feb 12;45(2):BSR20241133. doi: 10.1042/BSR20241133 (PMC12127793; doi:10.1042/BSR20241133)
Supplement: Figure S4 [file bsr-45-02-bsr-2024-1133-s004.docx]

**
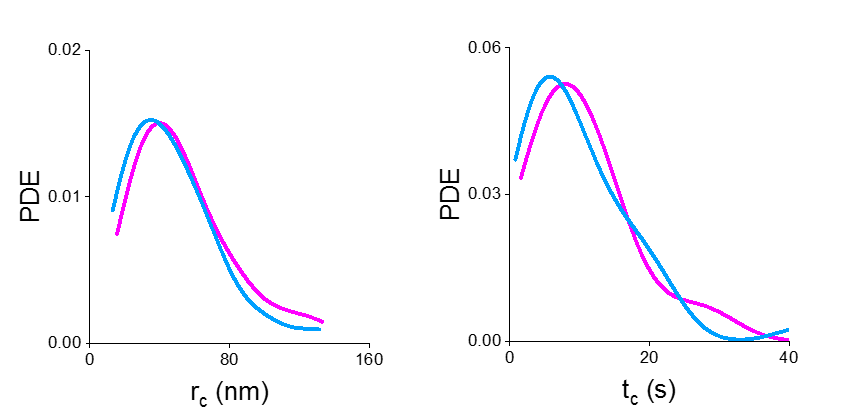
**

**Supplementary Figure S4.** Probability density estimate (PDE) for the corral radius (r_c_) and the characteristic time (τ_c_) obtained from the MSD_L_ analyses of perinuclear (magenta) and peripheral (light blue) filaments.
