# Supplementary material for: Dynamical organization of vimentin intermediate filaments in living cells revealed by MoNaLISA nanoscopy
Source: Biosci Rep. 2025 Feb 12;45(2):BSR20241133. doi: 10.1042/BSR20241133 (PMC12127793; doi:10.1042/BSR20241133)
Supplement: Figure S5 [file bsr-45-02-bsr-2024-1133-s005.docx]

**Supplementary Figure S5.** (**A**) Representative Airyscan image of a U2OS cell co-transfected with GFP-vimentin (green) and H2B-mCherry (magenta). Scale bar: 10 µm. (**B**) Analysis of filament´s curvatures by a Fourier decomposition method. The continuous line corresponds to the fitting of equation 4 to the Fourier data. The lp* value determined from Airyscan images was 2.0 ± 0.2 µm.
