# Supplementary material for: Dynamical organization of vimentin intermediate filaments in living cells revealed by MoNaLISA nanoscopy
Source: Biosci Rep. 2025 Feb 12;45(2):BSR20241133. doi: 10.1042/BSR20241133 (PMC12127793; doi:10.1042/BSR20241133)
Supplement: Figure S6 [file bsr-45-02-bsr-2024-1133-s006.docx]

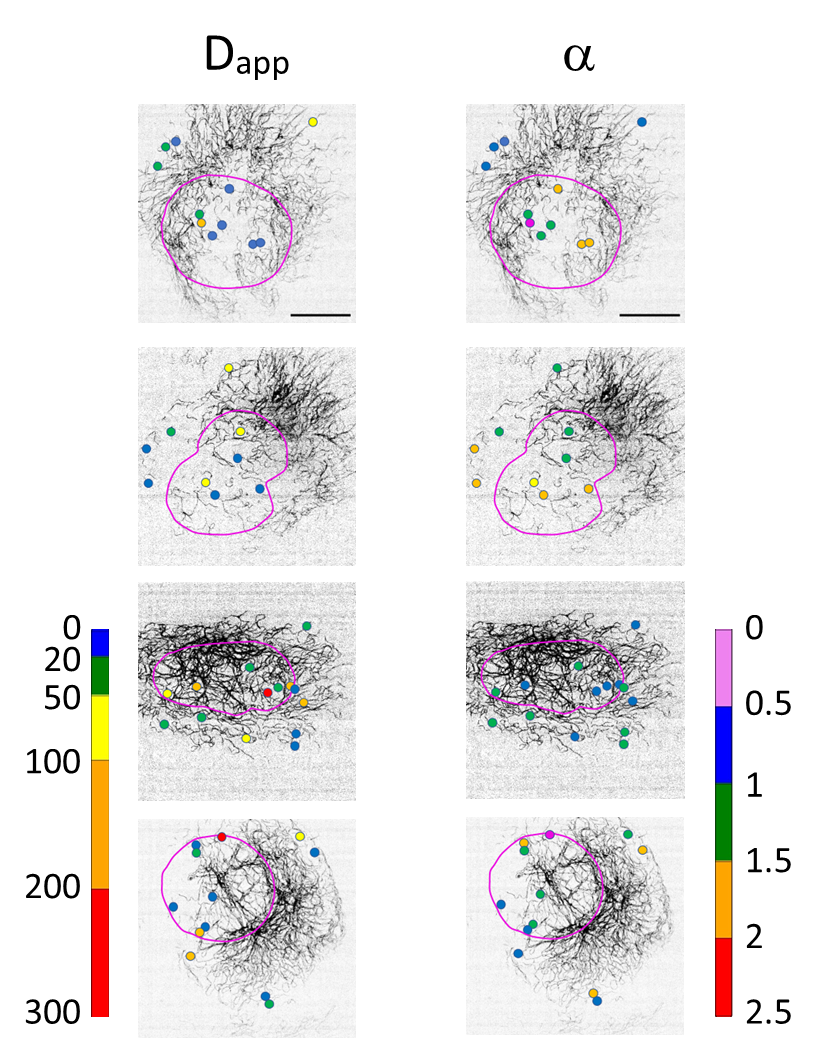


**Supplementary Figure S6.** Cellular maps of the parameters obtained from the anomalous diffusion model. D_app_ and α values obtained for the tracked filaments segments within individual cells were shown as color-coded dots located at the center of mass of each analyzed segment. Images were digitally saturated to simplify the visualization of the vimentin network (grayscale). The line (magenta) shows the contour of the cell nucleus. Scale bar: 10 μm.
