# Supplementary material for: Dynamical organization of vimentin intermediate filaments in living cells revealed by MoNaLISA nanoscopy
Source: Biosci Rep. 2025 Feb 12;45(2):BSR20241133. doi: 10.1042/BSR20241133 (PMC12127793; doi:10.1042/BSR20241133)
Supplement: Figure S7 [file bsr-45-02-bsr-2024-1133-s007.docx]

**
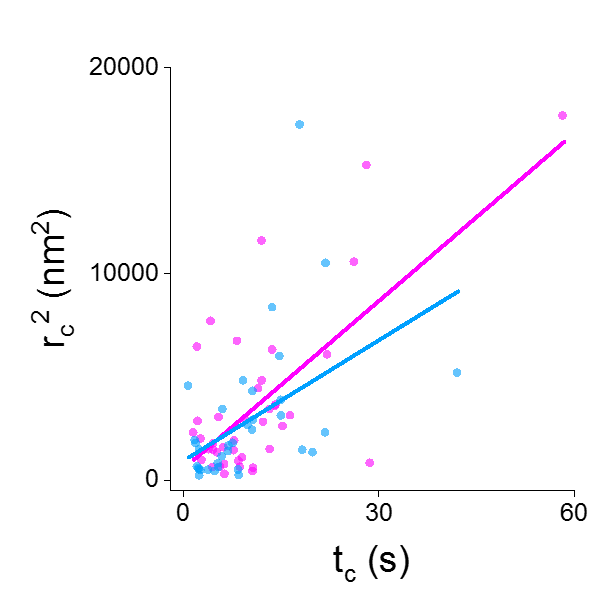
**

**Supplementary Figure S7.**  Estimation of the lateral diffusion coefficient (D) within corrals.  D was estimated considering the fitting parameters obtained from the MSD_L_ analysis of the experimental data (equation 6).  Parameters values recovered for perinuclear (magenta) and peripheral (light blue) filaments were fitted with a linear function derived from equation 7 obtaining D values of 130 ± 20 nm^2^/s and 97 ± 30 nm^2^/s, respectively.
