# Supplementary material for: Dynamical organization of vimentin intermediate filaments in living cells revealed by MoNaLISA nanoscopy
Source: Biosci Rep. 2025 Feb 12;45(2):BSR20241133. doi: 10.1042/BSR20241133 (PMC12127793; doi:10.1042/BSR20241133)
Supplement: Figure S8 [file bsr-45-02-bsr-2024-1133-s008.docx]

**Supplementary Figure S8.** Representative data obtained from the simulations of the lateral motion of vimentin filaments. (**A**) Trajectories and MSD_L_ data obtained for filaments in live cells (orange) and from the simulations (green). (**B**) Probability density estimate (PDE) for α and D_app_ values obtained by fitting the MSD_L_ data with the equation 8. To simplify the comparison between the experimental and simulated data, we pooled together the perinuclear and peripheral data.
