# Supplementary material for: Dynamical organization of vimentin intermediate filaments in living cells revealed by MoNaLISA nanoscopy
Source: Biosci Rep. 2025 Feb 12;45(2):BSR20241133. doi: 10.1042/BSR20241133 (PMC12127793; doi:10.1042/BSR20241133)
Supplement: Table S1 [file bsr-45-02-bsr-2024-1133-s009.docx]

| **Parameters** | **Value** | **Definition** |
| --- | --- | --- |
| D | 120 nm^2^/s | lateral diffusion coefficient within the corral |
| *I*_step_ | $\sqrt{2D dt}$ | step size within the corral |
| r_c_ | 10 - 60 nm | corral radius |
| τ_corral_ | 10 – 30 s | time interval between successive jumps |
| *l*_jump_ | 10 – 120 nm | jump size |
| *p*_jump_ | 0.5 - 1 | probability of jumps in the positive direction |

**Supplementary Table S1.** Set of parameters used for the numerical simulations.
